# Splenic Torsion Following Blunt Abdominal Trauma

**DOI:** 10.3390/jcm14145107

**Published:** 2025-07-18

**Authors:** Piotr Tomasz Arkuszewski, Agata Grochowska, Wiktoria Jachymczak, Karol Kamil Kłosiński

**Affiliations:** 1 Department of Biomedicine and Experimental Surgery, Faculty of Medicine, Medical University of Łódź, Narutowicza 60, 90-136 Łódź, Poland; 2 Students’ Scientific Association in Department of Biomedicine and Experimental Surgery, Medical University of Łódź, Narutowicza 60, 90-136 Łódź, Poland; agata.grochowska@student.umed.lodz.pl (A.G.); wiktoria.jachymczak@student.umed.lodz.pl (W.J.)

**Keywords:** splenic torsion, spleen trauma, blunt abdominal trauma, spleen avulsion, abdominal injury, splenic surgery, splenopexy, emergency surgery

## Abstract

**Background/Objectives**: Splenic torsion is a well-known and reported clinical problem. Splenic torsions after abdominal trauma represent a small group of cases that involve surgical management. They manifest primarily as abdominal pain, and the diagnosis is made based on imaging studies—ultrasound, CT, and MRI. **Methods**: This work aimed to analyze traumatic splenic torsions in terms of their clinical course, symptoms, timing, involvement of imaging techniques in the diagnosis, histopathological examination, and overall outcome. We searched databases using the desk research method under the keywords “splenic torsion”, “torsion”, and “spleen”, as well as in combination with “traumatic”, finding a total of eight cases, which we included in our analysis. **Results**: The eight cases were analyzed, comprising four females and four males, with an average age of 16.25 years (range 5–29 years). Traffic accidents were the most frequent cause of injury (five cases), while the circumstances were unclear in the remaining three. Immediate abdominal symptoms appeared in six patients. Splenic torsion was preoperatively diagnosed in five out of seven confirmed cases. A total of seven patients underwent laparotomy with splenectomy. In one case, laparoscopy converted to laparotomy with splenopexy preserved the spleen. Histopathology, performed in only two cases, confirmed splenic infarction in one patient; infarction status could not be determined in the remaining five due to missing data. **Conclusions**: Post-traumatic splenic torsions are a group of atypical injuries as the primary and immediate consequence of the trauma suffered is not anatomical–structural damage to the organ, such as a rupture. Mostly affecting young people, the cases described in the professional literature involve the main spleen, which was considered to be “wandering”, suggesting that this is a key predisposing factor for splenic torsion following blunt trauma and requiring diagnostic imaging for diagnosis.

## 1. Introduction

Spleen ruptures following blunt abdominal trauma are well-known in clinical practice. The primary cause of traumatic injuries, accounting for 50% to 75% of cases, is motor vehicle accidents [1,2]. Such post-traumatic splenic ruptures may be occult and not initially indicative of the presence of abdominal injuries [3]. In addition, they may manifest clinically a very long time after the blunt abdominal trauma was sustained, up to 2 years [4]. Laceration of the spleen following blunt trauma can also be caused by a fragment of a broken rib, and delayed splenic hemorrhage may be associated with left rib fractures [5,6]. Post-traumatic injuries to the spleen are not, however, limited to ruptures, lacerations, and tears of its parenchyma and capsule. Within this group, avulsion of the spleen, i.e., detachment of this organ from its blood vessels, should also be distinguished [7].

In addition to the post-traumatic splenic injuries mentioned above, a very small number of splenic torsions following blunt trauma have been described in the professional literature [8,9,10,11,12,13,14,15]. These are far fewer cases than those involving non-traumatic splenic torsion, which are widely and thoroughly analyzed [16].

This prompted the authors to analyze the cases of post-traumatic splenic torsion described in the professional literature. The aim of this study was to assess the mechanism of trauma, its temporal and causal relationship to splenic torsion, the age of the patients, the clinical manifestations, the diagnostic process, and the treatment methods and results.

## 2. Materials and Methods

A detailed, methodological research of the literature was conducted. The aim of this study was to analyze documented cases of post-traumatic splenic torsion to characterize their mechanism of origin, clinical course, diagnosis, and treatment outcomes.

### 2.1. Search and Study Selection Strategy

A comprehensive search of electronic databases was conducted between November 2024 and March 2025: PubMed, Scopus, Embase, ClinicalKey, Springer, Oxford Journals, BMJ Journals, Academic Search Ultimate (EBSCO), Elsevier Journals, Karger, and Wiley Online Library. An additional search was conducted in Google Scholar to identify publications not found in the main databases.

The combination of keywords and logical operators used were as follows: (“splenic torsion” OR “torsion” OR “spleen avulsion”) AND (“traumatic” OR “trauma” OR “blunt abdominal trauma”). No time or language restrictions were applied. In addition, the reference lists of included articles were manually searched for additional relevant studies.

The study selection process was conducted by the authors, who independently reviewed the titles and abstracts for potentially eligible articles. The same people then jointly assessed the full texts of the selected publications for compliance with the inclusion criteria.

The searches pooled a total of 8 studies. The systematic search in databases helped to identify 2 studies that were included, and citation searches also found 2 studies. Google Scholar helped identify the rest: two studies in English, one study in Polish that was not available in the databases, and one in the Chinese language.

### 2.2. Eligibility Criteria (PICOS)

Study inclusion and exclusion criteria were defined a priori using the PICOS (Population, Intervention, Comparison, Outcomes, Study Design) structure:

- Population (P): Patients of all ages with a diagnosis of splenic torsion occurring following blunt abdominal trauma;
- Intervention (I): Surgical treatment used, including laparotomy with splenectomy, laparoscopy with conversion to laparotomy, and splenopexy;
- Comparison (C): Due to the nature of this study being a case series analysis, a comparison group was not defined;
- Results (O): The endpoints analyzed included clinical course, symptoms’ characteristics, time from injury to diagnosis, role of imaging techniques (ultrasound, CT, MRI), intra-operative imaging, histopathology outcome, and final diagnosis and prognosis;
- Study Design (S): Only full-text case reports were included in the analysis.

The exclusion criteria were as follows: articles available only in the form of abstracts, papers of a review nature, cases of splenic torsion with a non-traumatic etiology, and no clear association with trauma.

In the manner described above, 8 cases meeting the given criteria were selected.

Due to the descriptive nature of this study—focusing on the collection and analysis of rare post-traumatic case reports rather than the evaluation of a clinical intervention—prior registration of this review in a database such as PROSPERO was not carried out as it was not appropriate for this type of project.

### 2.3. Data Extraction and Synthesis

Data from the eight included articles were extracted by the authors using a standardized form. The following information was collected for each case: age and sex of the patient, details of the injury, previous (pre-injury) diagnosis of wandering spleen, time and nature of onset of abdominal symptoms, preoperative diagnosis, intra-operative description of the organ, type of surgical procedure performed, histopathological result, and final post-operative diagnosis. In case of ambiguity in one of the publications, the authors were contacted directly for additional information [10]. The authors were also contacted for further information.

The collected data were qualitatively synthesized. A basic statistical analysis was also performed for the selected variables, calculating the arithmetic mean, standard deviation, and minimum and maximum values. The results were presented in descriptive and tabular form (Table 1 and Table 2).

It should be emphasized that this study was conducted on a relatively small group of cases and was primarily qualitative rather than quantitative in nature. Therefore, complex statistical calculations were not carried out, as with such a small study group, the results of these calculations would not be meaningful and statistically significant. The primary aim of this study was to expand knowledge of the issue under study and to develop recommendations useful in clinical practice.

## 3. Results (Clinical Characteristics of the Assembled Case Cohort)

The comprehensive literature search identified and collected a globally unique cohort of eight patients with documented post-traumatic splenic torsion. Analysis of this cohort enabled us to characterize this rare clinical phenomenon in terms of the circumstances of trauma, course, diagnosis, and treatment outcomes. Key observations from the synthesis of these cases are presented below (individual details are included in Table 1 and Table 2).

### 3.1. Patient’s Characteristics and Mechanism of Trauma

Analysis of the collected group of eight cases revealed a clear trend towards post-traumatic splenic torsion in young patients. The age of the patients ranged from 5 to 29 years (average 16.25 years, median 15.5 years), with an equal gender distribution (four females and four males). Both children and young adults were therefore affected. The predominant cause of injury, identified in 5 of the 8 cases, was traffic accidents of a different nature. It is noteworthy that in three cases, the exact circumstances of the abdominal trauma were not precisely defined in the original reports.

### 3.2. Clinical Picture and Time Course

A distinctive feature of the analyzed cohort was the obvious temporal association of symptoms with the trauma. In the vast majority of patients (6 of 8), complaints of abdominal pain occurred immediately after the incident. Importantly, these symptoms were not acute—requiring immediate intervention—but persisted or recurred, leading to further diagnosis. In one case, symptoms appeared with a two-day delay. This varied, and often a subacute clinical course is a key feature distinguishing post-traumatic splenic torsion from classic injuries such as splenic rupture.

### 3.3. Role of Diagnostic Imaging and Accuracy of Preoperative Diagnoses

Imaging studies played a key role in making the diagnosis. The high diagnostic success rate is noteworthy, as 5 of the 7 cases in which splenic torsion was finally confirmed had a correct preoperative diagnosis. One case was diagnosed with splenic fixation disorder in general, and the last case described an intra-abdominal hematoma and a conglomerate of twisted vessels, which contributed to the surgical treatment. Only in what appeared to be a splenic avulsion did imaging studies erroneously suggest the presence of an intra-abdominal abscess. These observations indicate that, although the clinical picture may be equivocal, modern imaging techniques can identify the pathology with a high degree of certainty, even if a precise diagnosis of torsion is not always possible before surgery.

### 3.4. Surgical Treatment Applied and Its Results

The predominant method of therapeutic management in the analyzed group was surgical intervention ending with splenectomy, which was performed in seven patients. In six of these cases, the immediate cause of organ removal was a twisting of its vascular pedicle, and in one case, an avulsion had already been performed. This highlights the radical nature of the treatment, most likely due to advanced ischemic changes at the time of surgery. However, it is worth noting one case in which the spleen was successfully preserved by performing laparoscopy with conversion to laparotomy and performing splenopexy. This is an extremely important observation that suggests that organ-sparing treatment is possible in selected cases. A major limitation in the assessment of the organ’s status was the lack of histopathological examination data in five cases after splenectomy, which prevented a clear assessment of the presence of splenic infarction. Infarction was confirmed in only one of the two cases examined. Notably, in all eight cases analyzed, the spleen was described as “wandering”, which seems to be a key predisposing factor for torsion after trauma.

In conclusion, the analysis of the collected group of cases allowed us to outline a consistent clinical picture of post-traumatic splenic torsion. It is a condition affecting mainly young people, often after traffic injuries, in whose pathogenesis an anatomical predisposition in the form of a “wandering spleen” appears to play a key role. The clinical picture, although often subacute, is effectively diagnosed by imaging studies. Splenectomy remains the predominant treatment modality, although a single case of successful splenopexy offers the possibility of spleen-sparing treatment. These key observations provide the starting point for a detailed discussion.

## 4. Discussion

Post-traumatic splenic torsions represent a rather specific group of post-traumatic splenic lesions, with characteristics different from splenic ruptures. This is because the direct and primary consequence of blunt abdominal trauma is not structural damage to the capsule or parenchyma of the organ but ischemia secondary to twisting of the splenic pedicle, causing impaired blood supply to the organ. The effects of ischemia become apparent at a later stage. This can be relatively short (hours and first days after injury) as well as longer (weeks after injury). Such splenic lesions are, however, an indirect consequence of the blunt abdominal trauma suffered earlier. Interestingly, the group of all described available cases, apart from being small, still has a relatively short history. Indeed, the oldest cases date only from the late 20th century. Meanwhile, the oldest and first available case in PubMed describing non-traumatic splenic torsion dates from the beginning of this century [17].

The clinical consequences of post-traumatic splenic torsion are not a uniform group of medical conditions. They are not limited only to the most expected ischemia or infarction of the organ but also include progressive and delayed avulsion. Furthermore, the clinical manifestation of these cases can occur soon after the patient’s injury as well as at a later stage. For this reason—as well as due to the rarity of such abdominal pathology and the lack of symptoms requiring immediate laparotomy associated with, for example, hemorrhage from an instantly ruptured or vascularly detached (avulsed) spleen—torsions of this organ represent a rather interesting group of sequelae of blunt abdominal trauma.

Bough et al., based on data from the literature and their own case, documented 409 cases of splenic torsion between 1888 and 2021 [16]. Against this background, the eight analyzed cases of splenic torsion following blunt trauma represent a very small group of this very rare abdominal pathology.

In the scientific material analyzed, it is noteworthy that all the cases searched and acquired are relatively new and come from a period when all the modern types of imaging studies were already known and available. The oldest is from 1997, and all cases were diagnosed before surgery based on modern imaging studies (USG, CT, and MRI), which undoubtedly facilitated therapeutic decisions. On the other hand, it is difficult to suppose that previously these types of cases were not encountered at all in clinical practice. The fact that the described cases come from the relatively recent past may be due to several factors. Certainly, in earlier years, the unavailability of advanced imaging studies made it difficult to diagnose post-traumatic splenic torsions and decide on surgical treatment. Consequently, patients’ intermittent and recurrent abdominal pain of undetermined etiology in the absence of a clear diagnosis may not have prompted doctors, as well as patients, to decide on surgical treatment. Chronic complaints that did not cause abdominal disease requiring immediate surgical treatment may have caused patients to become accustomed and adapt to the situation. If, on the other hand, surgery finally occurred after a long time, patients may no longer have remembered experiencing an abdominal trauma and may not have connected it with the ultimately diagnosed splenic torsion.

Another observation is the very different period from injury to diagnosis or surgical treatment. In four cases, it is a period calculated in weeks, and in the rest, it was less than a week. In the most extreme case, it was 2–3 months. Therefore, it is important to demonstrate or make plausible the connection of splenic torsion with blunt abdominal trauma, which in some cases occurred quite a long time ago. In this context, the appearance of symptoms soon after such a trauma and their persistence—as intermittent and recurrent complaints, for example—is significant. Particularly interesting is the case of a 19-year-old woman who eventually underwent surgical treatment, in which severe abdominal pain had been increasing for 3 days, and she gave a history of blunt abdominal trauma. In addition, she complained of nausea and vomiting and a body temperature elevated to 38.0 °C. Similar complaints, although of lesser severity, had occurred sporadically for 4 years. One year before her admission to the hospital, the patient had been hospitalized, and an abnormal position of the spleen was diagnosed at that time. However, only conservative treatment was undertaken at that time due to the lack of acute abdominal symptoms. This case demonstrates the severity and increased frequency of symptoms in a patient with a previously diagnosed wandering spleen.

In some cases, the intermittent and recurrent nature of the complaints can be explained by torsion and detorsion of the spleen and its wandering in the abdominal cavity. This may be a consequence of post-traumatic stretching or partial or complete rupture of the splenic ligaments. However, it should be noted that, in some cases, the splenic ligaments were not found at all and it was concluded to be a wandering spleen. In these situations, there may have been a post-traumatic rupture of the splenic ligaments. If, on the other hand, the lack of ligaments was congenital, then blunt abdominal trauma could have put the prone spleen into excessive mobility. Another possible situation was that the previously ligamentless spleen only changed position after avulsion [10]. All cases involving the main spleen were considered by the authors of the respective descriptions to be a wandering spleen, which by definition is predisposed to torsion. In this regard, it is worth noting a case in which a splenic infarction and concomitant pancreatic torsion occurred 5 years following abdominal trauma from a car accident. However, the current complaints had lasted for a week, and therefore, it was difficult to recognize the existence of a link between the trauma and splenic torsion [18].

Despite the small study group, it is possible to conclude that there is no close correlation between the presence of splenic infarction and the short period from injury to surgery. In one case, in which surgery occurred 3 days after the injury, no splenic infarction was diagnosed, although the patient had been diagnosed with an anomalous splenic position a year earlier [11]. In another case, surgery occurred similarly early (3 days after injury), although splenic infarction was later confirmed by histopathology [15]. In contrast, even 3 months after the injury and despite the splenic pedicle being twisted three times, it was possible to perform a successful splenopexy, thus ruling out splenic infarction [14]. This shows that even several weeks after injury and the onset of complaints, there are chances of splenic preservation.

An important observation is the relatively mild clinical course of post-traumatic splenic torsion, which differs significantly from the typical post-traumatic splenic injuries of rupture and avulsion. In the analyzed material, the patients did not present symptoms of hemorrhagic shock and did not require surgery for vital indications, although a decrease in blood hemoglobin concentration was observed in the patient with avulsion. Most of these cases were relatively mild injuries that did not necessitate immediate surgical intervention, nor were they associated with the development of other significant abdominal injuries.

Also of note is the young age of the patients, up to 29 years. This observation is consistent with views that splenic injuries are more common in children than in adults [19]. In this study, children (under the age of 18) account for 5 of the 8 cases included in the tables (Table 1). Due to the anatomical differences between an adult and a child, children are predisposed to splenic injuries. Normally, in an adult, the spleen is usually anchored in the left upper quadrant by three ligaments, namely the gastrosplenic, splenorenal, and phrenicocolic ligaments, and it is tucked under the rib arch, which provides additional protection for it. The rib arch in infants and young children does not completely cover the spleen. In addition, in children, the fat and muscle tissue are not as developed as in adults. These differences may make the spleen in children particularly vulnerable to blunt abdominal trauma [20]. It is interesting to note that the cases of rupture of the wandering spleen following blunt trauma described in the literature also involve young people, 5 years and 31 years [21,22]. In addition, splenic wandering itself occurs symptomatically at two age peaks: (1) in children under 10 years of age; and (2) in women of childbearing age [23]. In the material studied, the children were 5, 9 (in two cases), 14, and 17 years old, while among the adults, there were two women aged 19 and 29 years and a man aged 28 years. This indicates a tendency for post-traumatic splenic torsion to occur precisely in children and young adults.

The radiological signs found in patients with traumatic splenic torsion are varied. Among the most important are displacement of the spleen beyond its typical location, a spiral/corkscrew/coil/whirled appearance sign, a lack of contrast enhancement, and a lack of splenic perfusion.

A case of an atypical course of post-traumatic splenic torsion described by Kim et al. deserves a separate discussion. It concerned an avulsion of the wandering spleen following a previous post-traumatic torsion. A 5-year-old boy had a pedestrian injury. Initially, CT showed a small amount of intraperitoneal fluid and a poorly enhanced splenic avulsion in the left upper quadrant. Based on this, the authors concluded that a splenic torsion was already present. In a direct exchange of information, one of the authors indicated that a complete avulsion of the spleen occurred following a prolonged torsion of its vessels and that the spleen was subsequently displaced into the right paracolic gutter, which was suggested by a subsequent CT scan and definitively confirmed intra-operatively. In this author’s opinion, the splenic infarction was the result of both vascular torsion and vascular disruption. It should be noted that, in this case, the avulsion of the spleen was not the result of its rapid and sudden movement with displacement immediately following the blunt abdominal trauma but arose later as a result of prolonged splenic torsion. Only the complete detachment of the spleen from the vessels resulted in its movement to another region of the abdomen. The author further pointed out that intra-operatively, the spleen was in a “wandering” state and showed no signs of splenic ligaments. Since the first CT showed the presence of the organ in the left upper quadrant and the intra-operatively avulsed spleen was found in the right paracolic gutter, it can be concluded that despite the absence of ligaments, the spleen was maintained in its proper position as long as the continuity of its vessels was preserved.

One case of post-traumatic torsion of the accessory spleen has been described in the literature [24]. A 12-year-old boy suffered abdominal trauma as a result of a beating to the left abdomen. He complained of recurrent pain in this part of the abdomen. An abdominal CT scan showed an oval mass in the upper left abdomen adjacent to the pancreatic tail, clearly separated from the spleen, the left kidney, and the left adrenal gland. Based on imaging studies (CT, ultrasound), torsion of the vascular pedicle of the accessory spleen was diagnosed. Twenty-five days after the injury, laparoscopic resection was performed, and the accessory spleen was found to adhere to the omentum and colon, twisted four times around its axis. The cut surface of the excised specimen showed red splenic tissue, and pathological examination revealed hemorrhagic infarction of the accessory spleen, confirming the diagnosis of accessory spleen torsion. The time followed by laparoscopy—25 days—was much shorter than in the only analyzed case of post-traumatic splenic torsion, in which the spleen was successfully preserved; that case took 3 months, and the pedicle was twisted three times. Time is not a criterion for determining whether splenic infarction has occurred, and the degree of twisting of the vascular pedicle may be decisive.

It is worth noting that splenic torsion can cause various other abdominal and urinary pathologies (e.g., painful splenomegaly or urinary retention) or can imitate other acute abdominal diseases [25,26,27,28]. Such surgical conditions could theoretically also be an indirect and distant consequence of blunt abdominal trauma resulting in splenic torsion.

The main treatment for splenic torsion and post-traumatic splenic ruptures is splenectomy, although spleen-tissue-sparing surgical procedures can be successfully used in some cases [16,29]. In all analyzed cases of post-traumatic splenic torsion, surgical treatment was carried out. It appears to be unavoidable in any form, even when minimally invasive. This is because undertaking such treatment allows, first and foremost, the viability of the spleen to be assessed. If the spleen’s blood supply returns after detorsion, splenopexy is necessary. In other cases, on the other hand, surgical intervention and splenectomy prove essential as they avoid the later consequences of the presence of a necrotic spleen in the abdominal cavity.

Post-traumatic splenic torsions are a group of relatively similar cases characterized primarily by the appearance of abdominal discomfort soon after blunt abdominal trauma and symptoms of twisting of the splenic vascular pedicle. In an extreme situation, the consequence of traumatic torsion of the spleen may be its avulsion. On the other hand, symptoms may persist for a long time before a decision on surgical treatment is made (even several weeks).

Each of the cases analyzed was considered to be a wandering spleen. This means that such a hypermobile spleen is particularly susceptible to being set in motion and rotated with twisting of the vascular pedicle following blunt abdominal trauma.

### Limitations of This Study

It should be emphasized that the present analysis is based on a small number of eight case reports from different centers and time periods. The data contained in the original publications were sometimes incomplete, particularly concerning the histopathological findings. Furthermore, the retrospective nature of this study makes it impossible to draw conclusions on causal relationships with the same certainty as in prospective studies.

## 5. Conclusions

An important observation from this study is that post-traumatic splenic torsions are a group of “atypical” injuries. This is because the primary and immediate consequence of the blunt abdominal trauma suffered is not structural damage to the organ, such as rupture, but an anatomical displacement reconfiguration consisting of a twisting of the splenic vascular pedicle. This, in turn, leads to the loss of the spleen as a result of an ischemic process or avulsion but not following “mechanical” damage to the parenchyma of this organ itself. Therefore, a more appropriate term for this type of case would be “post-traumatic condition” rather than “injury”. Another observation is that all cases of post-traumatic splenic torsion described in the professional literature involve a spleen that has been found to be a “wandering spleen”. This clearly indicates that only such a hypermobile spleen is susceptible to torsion of its vascular pedicle after suffering blunt trauma. Moreover, post-traumatic splenic torsion affects young people, mainly children, adolescents, and young adults, and is analogous to post-traumatic splenic ruptures. None of the analyzed cases resulted in the death of the patient. Therefore, traumatic splenic torsion, although rare, does not represent a clinical problem in the sense that it is diagnosed on the basis of imaging studies or allows the indication for surgical treatment to be determined, even in the absence of an accurate and precise preoperative diagnosis.

Therefore, in young patients diagnosed with a wandering spleen who report persistent or recurrent abdominal pain following blunt trauma, a high index of suspicion for vascular pedicle torsion should be maintained to allow early diagnosis and potential organ-sparing treatment.

## Figures and Tables

**Table 1:** All the analyzed cases of splenic torsion following blunt abdominal trauma—part 1.

Author, Year, and Citation	Sex	Age	Details of the Trauma	Previous Diagnosis (Before Trauma) of Wandering Spleen	Abdominal Symptoms Immediately After Trauma	Duration of Symptoms from Trauma to Initial Diagnosis
Walcher, 1997 [8]	F	29	Motor vehicle crash (a driver of a car)	−	+ (Recurrent symptoms)	8 weeks
Raissaki, 1998 (case one of three) [9]	M	28	Unspecified blunt abdominal trauma	−	+ (Aggravating)	3 weeks
Kim, 2003 [10]	M	5	Pedestrian injury	−	+ (Aggravating)	5 days
Molski, 2005 [11]	F	19	Unspecified blunt abdominal trauma	+	+ (Aggravating)	3 days
Saouab, 2010 [12]	F	9	Road accident	+	+ (No data on the dynamics of symptoms)	Immediate symptoms after the trauma
Kaabar, 2014 [13]	F	17	Unspecified abdominal trauma	+	? (No precise data available)	At least 4 days
Zavidic, 2020 [14]	M	9	Bicycle crash	Spleen fixation disorder, no previous diagnosis of a wandering spleen	+ (Persistent)	2 months
Wang, 2023 [15]	M	14	Lower abdominal trauma (hit against a bicycle handlebar)	−	− (Onset 2 days after the trauma, recurrent)	1 day

**Table 2:** All the analyzed cases of splenic torsion following blunt abdominal trauma—part 2.

Author, Year, and Citation	Preoperative Diagnosis	Intra-Operative Appearance	Surgical Procedure	Infarction on Histopathological Examination	Postoperative Diagnosis
Walcher, 1997 [8]	Torsion of the spleen (lack of perfusion based on ultrasound, MRI, and angiography).	The spleen twisted clockwise 180° and was attached at its hilum by a long pedicle. The organ was devoid of its usual peritoneal ligaments. After untwisting, the spleen showed persistent ischemia.	Laparotomy, splenectomy	− (not confirmed on HP)	Torsion of the wandering spleen
Raissaki, 1998 (case one of three) [9]	Torsioned splenic pedicle (based on CT—spiral sign).	An intrapelvic torsioned wandering spleen containing areas of contusion and hematoma caudally.	Laparotomy followed by splenectomy	No data	Torsion of the wandering spleen
Kim, 2003 [10]	Suspicion of intra-abdominal abscess (based on CT, there was no leakage of contrast).	Spleen avulsed into the right paracolic gutter and infarcted with preservation of its capsule, hematoma in the right paracolic gutters, short avulsed vascular pedicle at the pancreatic tail, no evidence of gastric, peritoneal, or diaphragmatic attachment of the spleen in the left upper quadrant.	Laparotomy, splenectomy	No data	Avulsion of wandering spleen following traumatic torsion
Molski, 2005 [11]	Torsion with ischemia of the displaced spleen (based on CT, a wandering spleen was previously diagnosed).	Splenic pedicle torsion, splenic vein thrombosis, organ ischemia, and linear rupture of the splenic capsule. Complete lack of splenic ligaments.	Laparotomy with splenectomy	No data	Torsion of the wandering spleen
Saouab, 2010 [12]	Torsion of the wandering spleen. Spleen at the periumbilical region, hypodense, not enhanced after contrast injection due to torsion. There is another round mass adjacent, which is composed of vascular structures surrounded by fat in a characteristic ”whorled appearance”.	No details.	Laparotomy, splenectomy	No data	Torsion of the wandering spleen
Kaabar, 2014 [13]	Acute torsion with infarction of the wandering spleen.	A pelvic, necrotic, twisted spleen around the pedicle without ligaments.	Laparotomy, splenectomy	No data	Torsion and necrosis of the wandering spleen
Zavidic, 2020 [14]	Spleen fixation disorder.	Triple torsion of the splenic artery and vein.	Laparoscopy converted to laparotomy, splenopexy	No data	Torsion of the wandering spleen
Wang, 2023 [15]	Intra-abdominal hematoma, an intra-abdominal cluster of blood vessels coil (based on CT and abdominal puncture).	The spleen, missing from its normal location, was found in the omentum. Spleen with a 720° twist; 4 cm rupture in the spleen.	Laparotomy, splenectomy	+ (confirmed on HP)	Wandering spleen torsion and rupture bleeding

## Data Availability

The original contributions presented in this study are included in the article. Further inquiries can be directed to the corresponding author.
